# Trajectories of Blood Pressure in Patients with Established Coronary Artery Disease over 20 years

**DOI:** 10.1155/2022/2086515

**Published:** 2022-08-05

**Authors:** Piotr Jankowski, Paweł Kozieł, Grzegorz Bilo, Jarosław Pinkas, Danuta Czarnecka, Kalina Kawecka-Jaszcz, Andrzej Pająk

**Affiliations:** ^1^Department of Internal Medicine and Geriatric Cardiology, Centre of Postgraduate Medical Education, Warsaw, Poland; ^2^Department of Epidemiology and Health Promotion, School of Public Health, Centre of Postgraduate Medical Education, Warsaw, Poland; ^3^Department of Medicine and Surgery, University of Milano-Bicocca, Milan, Italy; ^4^Department of Cardiology, IRCCS, Istituto Auxologico Italiano, Milan, Italy; ^5^School of Public Health, Centre of Postgraduate Medical Education, Warsaw, Poland; ^6^I Department of Cardiology, Interventional Electrocardiology and Hypertension, Institute of Cardiology, Jagiellonian University Medical College, Kraków, Poland; ^7^Department of Epidemiology and Population Studies, Institute of Public Health, Faculty of Health Sciences, Jagiellonian University Medical College, Kraków, Poland

## Abstract

**Objective:**

To evaluate changes in blood pressure (BP) values in patients with established coronary artery disease (CAD) over 20 years (1997–2017).

**Materials and Methods:**

Consecutive patients aged <71 years and hospitalized for acute coronary syndrome or myocardial revascularization procedures were recruited and interviewed 6–18 months after their discharge from the hospital. BP was measured in 1997–1998, 1999–2000, 2006–2007, 2011–2013, and 2016–2017. The same five hospitals took part in the surveys at each time point.

**Results:**

We examined 412 patients in 1997–1998, 427 in 1999–2000, 422 in 2006–2007, 462 in 2011–2013, and 272 in 2016–2017. The proportion of patients with BP at the recommended goal was 49.2% in 1997–98, 44.5% in 1999–2000, 44.7% in 2006–07, 51.1% in 2011–13, and 58.8% in 2016–17 (*p* < 0.001). Mean systolic and diastolic BP decreased significantly independent of age, sex, and education (systolic BP: 137.9 ± 21.4 mmHg in 1997–98, 139.5 ± 21.6 mmHg in 1999–2000, 136.1 ± 20.3 mmHg in 2006–07, 134.8 ± 22.0 mmHg in 2011–13, and 134.2 ± 18.6 mmHg in 2016–17, *p* < 0.001; diastolic BP: 83.4 ± 11.0 mmHg in 1997–98, 84.8 ± 12.0 mmHg in 1999–2000, 85.2 ± 11.0 mmHg in 2006–07, 80.9 ± 12.5 mmHg in 2011–13, and 81.1 ± 10.4 mmHg in 2016–17; *p* < 0.001).

**Conclusion:**

The analysis of five multicenter surveys provides evidence of a decrease in BP in patients with established CAD over two decades. This trend is independent of age, sex, and the education level of the patients.

## 1. Introduction

Cardiovascular disease is the leading cause of death in developed countries [1]. Despite advances in the pharmacological and invasive treatment of coronary artery disease (CAD) in recent years, mortality following myocardial infarction remains high [2]. The main causes include inadequate control of risk factors, insufficient lifestyle changes, and suboptimal pharmacotherapy [3, 4]. Recently published results from Europe and North America showed that about half of all CAD patients have their blood pressure (BP) over 140/90 mmHg [3, 5–7]. The current guidelines strongly recommend a BP reduction in patients with CAD and hypertension [8, 9]. The current optimal BP values for patients with established cardiovascular disease have been a subject of debate for decades [10–13]. The present analysis aimed to assess trends in BP values in patients with established CAD over 20 years (1997–2017).

## 2. Methods

We analyzed the data of the participants of the five surveys assessing cardiovascular prevention following hospitalization due to CAD that were conducted in 1997–1998 (survey I), 1999–2000 (survey II), 2006–2007 (survey III), 2011–2013 (survey IV), and 2016–2017 (survey V). The same five hospitals serving the city and surrounding districts (1.2 m inhabitants) participated in each survey. The methods used in these surveys had been published previously and were similar on each occasion [5, 14–17]. In brief, the study sample in each survey consisted of consecutive patients hospitalized for myocardial infarction, unstable angina, percutaneous coronary intervention, or coronary artery bypass grafting. Since the age limit in the first (1997–1998) and second (1999–2000) surveys were <71 years we excluded from the present analysis all older participants participating in the other three surveys.

The patients were examined at 6–18 months after the index hospitalization. Data on patients' medical history and the medications used were obtained using a standard questionnaire. Education of the participants was assessed based on the number of years of schooling as well as a categorical variable (at least secondary education vs. lower than secondary education). Height and weight were measured in a standing position without shoes and heavy outerwear using standard scales with a vertical ruler. The scales were calibrated at the start of each survey. Body mass index was calculated according to the following formula: body mass index = weight (kg)/(height (m))^2^.

Blood pressure was measured twice, on the right arm in a sitting position after at least five minutes of rest. The average of the two readings was used in the analysis. High blood pressure was defined using two approaches. First, we analyzed the proportions of patients achieving recommended BP (according to the European Society of Hypertension/European Society of Cardiology guidelines) at the time of each survey (BP goal of <140/90 mmHg (<130/80 mmHg in diabetics) in 1997–1998, 1999–2000, 2006–2007 and 2011–2013; <140/90 mmHg (<140/85 mmHg in diabetics) in 2016–2017) [8, 18–21]. The second definition of high pressure included all cases of systolic BP at least 140 mm of Hg or diastolic BP at least 90 mm of Hg. Participants were considered hypertensive if they had high blood pressure (≥140/90 mm of Hg) during the follow-up interview or if they were treated with antihypertensive drugs at that time and had been diagnosed with hypertension during index hospitalization [22]. The patients were considered to have undiagnosed hypertension if they had hypertension (as defined above) that has not been diagnosed during the index hospitalization. In addition, self-reported use of BP-lowering drugs was analyzed.

The survey protocols were approved by the institutional Bioethics Committee. Every patient signed an informed consent form.

### 2.1. Statistical Analysis

Categorical variables were reported as percentages and continuous variables as means with standard deviations (SD). The Pearson *χ*^2^ test was applied to all categorical variables. Normally distributed continuous variables were compared by the Student's *t*-test or analysis of variance. Variables without normal distributions were evaluated by the Mann–Whitney *U* test or the Kruskal–Wallis analysis of variance. Temporal trends were evaluated with linear regression for continuous variables and logistic regression for categorical variables with subsequent surveys coded as an independent variable. Multivariable logistic regression analysis was used to calculate the odds ratios of having BP at goal in surveys II, III, IV, and V compared to the first survey. As a sensitivity analysis, we used two definitions of high blood pressure (see above). In addition, we performed the analysis limited to patients with hypertension only. Generalized linear models as implemented in the Statistica 13 software (TIBCO Software, Palo Alto, USA) were used to provide the least square means after adjusting for the covariates including sex, age, education, and employment, index event, and body mass index. A two-tailed *P* value of less than 0.05 was regarded as indicating statistical significance.

## 3. Results

The following numbers of patients participated in the surveys: 415 in 1997–1998, 427 in 1999–2000, 421 in 2006–2007, 456 in 2011–2013, and 274 in 2016–2017. The characteristics of the studied groups are presented in Table 1. The participants of the fifth survey were older, better educated, and more often in employment compared to the participants of the first survey. No significant differences were observed in sex distribution between the surveys. On the other hand, the mean number of BP-lowering drugs increased significantly (Table 1). At least one BP-lowering drug was used by 85.5% of the patients in 1997–1998, 88.8% in 1999–2000, 97.4% in 2006–2007, 93.0% in 2011–2013, and 99.3% patients in 2016–2017. *β*-blockers were used by 59.0%, 63.9%, 87.4%, 80.7%, and 92.7% of the patients, angiotensin-converting enzyme inhibitors by 45.8%, 47.5%, 74.5%, 66.2%, and 74.8%, angiotensin receptor blockers by 0.0%, 0.0%, 5.3%, 12.5%, and 14.2%, calcium antagonists by 28.7%, 33.3%, 21.0%, 20.6%, and 27.7%, diuretics by 17.1%, 20.1%, 31.8%, 35.8%, and 41.2%, whereas other antihypertensive drugs by 0.5%, 2.1%, 2.4%, 6.8%, and 2.9% of the participants of the survey I, II, III, IV, and V, respectively.

**Figure 1:**
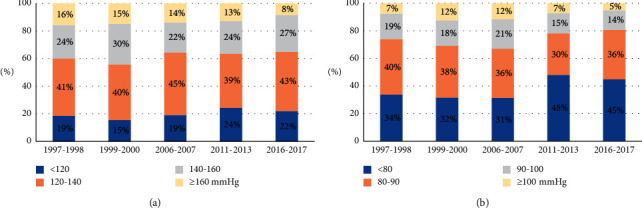
Proportion of patients by systolic (a) and diastolic (b) blood pressure category and survey.

Figure 1 presents the systolic and diastolic BP categories by the survey. During the observation period the mean systolic BP, diastolic BP, and mean arterial pressure values decreased significantly (Table 2). However, this trend was presently starting from the third survey onwards when systolic BP was taken into the account and from fourth survey onwards when diastolic BP was considered. These findings were confirmed by a comparison of the adjusted means of systolic (139.3 (20.8) mmHg in 1997–1998 vs. 140.1 (20.5) mmHg in 1999–2000 vs. 135.8 (20.3) mmHg in 2006–2007 vs. 134.0 (20.4) mmHg in 2011–2013 and 132.9 (21.1) mmHg in 2016–2017, *p* < 0.001) and diastolic BP (83.3 (11.7) mmHg in 1997–1998 vs. 84.7 (11.6) mmHg in 1999–2000 vs. 85.1 (11.5) mmHg in 2006–2007 vs. 80.9 (11.5) mmHg in 2011–2013 and 81.5 (11.9) mmHg in 2016–2017, *p* < 0.001). These trends were independent of age, sex, and education of the participants (Table 3). The trends were significant in patients without diabetes (systolic BP *p* < 0.001, diastolic BP *p* < 0.001) and in patients with diabetes when systolic BP (*p* < 0.001), but not diastolic BP (*p*=0.35) was analyzed.

The criteria for hypertension were met by 67% of the patients in 1997–1998, 73.1% in 1999–2000, 87.5% in 2006–2007, 81.6% in 2011–2013, and 86.9% in patients studied in 2016–2017 (*p* < 0.001). The multivariable adjustments did not change the results significantly. Proportion of hypertensive patients without diagnosis of hypertension during the index hospitalization was 13.5% in 1997–1998, 11.2% in 1999–2000, 6.5% in 2006–2007, 6.6% in 2011–2013, and 2.9% in 2016–2017 (*p* < 0.001). The multivariable adjustments did not change the results significantly.

**Figure 2:**
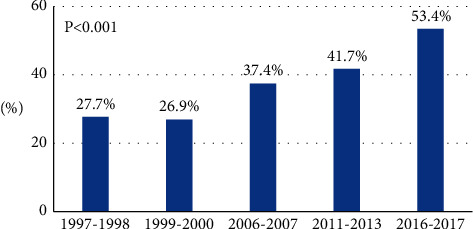
Proportion of hypertensive patients with blood pressure at goal by survey.

The proportions of patients with BP at recommended goal increased significantly from 49.2% in 1997–98 to 58.8% in 2016–17 during the 20-year timeframe of the study (Table 2). The increase was independent of co-factors and was visible starting from the fourth survey (Table 4). An even greater increase was shown when we limited the analysis to patients with hypertension only (from 27.7% in 1997–1998 to 53.4% in 2016–2017; Figure 2).

## 4. Discussion

There is strong scientific evidence that the long-term survival of coronary patients may be improved by providing optimal cardiovascular prevention [8, 9]. Indeed, according to the recently published results of the EUROASPIRE V survey, there is a considerable potential for further improvement in cardiovascular risk in patients with CAD [3]. Our results showed a decrease in BP in patients with established coronary artery disease in the 21st century. Such trend paralleled the mean number of BP-lowering agents prescribed and was evident beginning from 2006 when regarding systolic BP and from 2011 when diastolic BP was considered. These findings were confirmed by multivariate analyses. Despite a favorable trend in the proportion of patients with high BP, BP values remained high in 41% of the most recent survey participants. This suggests a great potential for further reduction in cardiovascular risk in patients with hypertension through better management of the condition. Although we could not compare data on the lifestyle of all survey participants, the increase in the mean body mass index may suggest that patients with established CAD have inappropriate lifestyle habits, including diet and physical activity [23]. The present results point to improvement in cardiovascular prevention in patients with coronary artery disease.

We found an increasing prevalence of hypertension in patients with CAD. This may reflect the trends present in the general population of Poland [24]. The proportion is higher than in the general population (in the case of the Polish general population 33–43% of subjects are hypertensive [24, 25]) since hypertension is a risk factor for CAD and since the average age of our population was relatively high. The estimated prevalence of hypertension may be associated with an error related to different criteria of hypertension diagnosis that were applied by different hospitals participating in the study. The present results may also have been influenced by variation in BP levels, which might have led to patients being misclassified as hypertensive/normotensive.

Elevated BP and/or the intake of BP-lowering drugs are used for hypertension diagnosis in many studies assessing the prevalence of hypertension in the general population [24, 26]. These studies did not consider the errors associated with the intake of BP-lowering drugs for reasons other than hypertension (angina, heart failure, history of myocardial infarction, peripheral artery disease) because the prevalence of these conditions is much lower than the prevalence of hypertension. In the case of the present study population, this definition could not be applied since 92.3% of the subjects were taking at least one drug with BP-lowering potential. On the other hand, a diagnosis of hypertension on discharge from the hospital did not qualify a patient as being hypertensive or normotensive during the follow-up examination since 30.3% of patients without a diagnosis of hypertension during hospitalization had a BP level of at least 140/90 mmHg 6–18 months after discharge, thereby classifying them as hypertensive. Indeed, a physician's diagnosis of hypertension should be treated with caution when used to identify CAD patients with hypertension [22].

The definition of hypertension used in this analysis does not include patients with a history of elevated BP (or even with a hypertension diagnosis) and who had normal (<140/90 mm of Hg) BP during the control visit and who were not taking antihypertensive medication. Similarly, such patients were not considered hypertensive in other large epidemiological studies [24, 26, 27]. Subjects with elevated BP during hospitalization but without a diagnosis of hypertension at discharge were considered to be hypertensive if their BP was ≥140/90 mmHg during the follow-up visit. If, however, the BP of these subjects was below 140/90 mm of Hg it can be reasonably assumed that no diagnosis of hypertension at discharge was correct. This is because elevated BP values in patients with acute coronary episodes or during the period close to percutaneous or surgical coronary intervention do not always reflect actual hypertension in everyday conditions and should not be used as a basis for diagnosing hypertension. The definition of hypertension used in the present study does not apply to the subjects whose discharge letters did not include a hypertension diagnosis and who during the control visit were on antihypertensive medication and had normal BP levels. It can thus be assumed that the percentage of patients with CAD and undiagnosed hypertension may be higher than the present findings suggest.

In general, there are three reasons for poor BP control: the patient, the physician, and the healthcare system. The patient may not adhere to the medication regiment or may be resistant to it. As the mean number of antihypertensive drugs was lower in those achieving the BP goal (1.9 ± 1.0 vs. 2.1 ± 1.0), this may indicate lower compliance with medication in those not achieving the BP goal or that their hypertension was somewhat resistant to treatment. Low awareness of high BP as an important cardiovascular risk factor may also be one of the reasons. Low medication persistence is considered a major factor limiting the long-term effectiveness of antihypertensive treatment [28]. A quite frequent cause of poor BP control is clinical inertia [29]. When physicians are asked what they should do to combat inadequate BP control, they usually respond with the correct answer (increase the dosage, add an antihypertensive drug, or switch to another antihypertensive drug). However, when this issue is observed in a real-world setting, no action is taken in many cases [30]. The healthcare system may also be partially responsible. This may be due to a low drug reimbursement rate, different rules for antihypertensive drug reimbursement, time constraints, the poor working conditions of physicians and nurses, etc.

Numerous studies have assessed the quality of medical care in connection with prevention and treatment of CAD, including the control of hypertension [3, 6, 7, 31]. An analysis of these data suggests that a high proportion of CAD patients have BP levels above the recommended goal in most European countries. Although most patients receive BP-lowering drugs, a large proportion of them have elevated BP values. To the best of our knowledge, the present study is the first to present data allowing to estimate the operational efficacy of the same hospitals in terms of secondary prevention of CAD events over a 20 year observation period. This provides a unique opportunity to track changes in the BP control of patients with CAD living in a specific area over a long period and who were treated in the same hospitals.

### 4.1. Limitations of the Study

Our analysis has some limitations. Firstly, we were unable to assess the influence of changes in BP on the risk of cardiovascular complications. Secondly, participants were limited to those who had experienced an acute CAD event or had undergone a revascularization procedure. Therefore, our results should not be directly applied to other CAD patients. Thirdly, the survey participants lived in a defined geographical area. Although the applicability of our results to other regions is uncertain, the trends described above reflect changes over time in the general population in Poland as well as in CAD patients from other European countries [24, 31]. However, an important advantage of our analysis is that our results are not based solely on abstracted medical record data but took into account face-to-face interviews and examinations using the same protocol and standardized methods and instruments. Therefore, to best of our knowledge, the presented results provide the most up-to-date and reliable information on BP trajectories in patients with CAD over the last 20 years.

## 5. Conclusion

The analysis of five multicenter surveys provides evidence of a decrease in BP in patients with established CAD over a period of two decades. The trend is independent of age, sex, and the education level of patients.

## Figures and Tables

**Table 1:** Characteristics of the study group by the survey.

	Survey I *n* = 415	Survey II *n* = 427	Survey III *n* = 421	Survey IV *n* = 456	Survey V *n* = 274	*p* for trend
Age, years, mean (SD)	57.9 (8.3)	58.6 (8.1)	59.9 (7.5)	61.1 (6.9)	62.6 (6.9)	<0.001
Sex						
Men, *n* (%)	303 (73.0)	298 (69.8)	300 (71.3)	309 (67.8)	204 (74.5)	0.79
Women, *n* (%)	112 (27.0)	129 (30.2)	121 (28.7)	147 (32.2)	70 (25.5)	
Duration of education, years, mean (SD)	11.4 (3.6)	11.6 (3.5)	11.9 (3.3)	12.1 (3.1)	13.0 (3.1)	<0.001
Employed, *n* (%)	71 (17.1)	75 (17.6)	154 (36.6)	122 (26.9)	121 (44.2)	<0.001
Index event						
Myocardial infarction, *n* (%)	114 (27.5)	115 (26.9)	98 (23.2)	154 (33.8)	95 (34.7)	0.007
Unstable angina, *n* (%)	94 (22.7)	95 (22.3)	112 (26.6)	142 (31.1)	47 (17.2)	0.56
PCI, *n* (%)	99 (23.9)	101 (23.7)	134 (31.8)	113 (24.8)	116 (42.3)	0.001
CABG, *n* (%)	108 (26.0)	116 (27.2)	77 (18.3)	47 (10.3)	16 (5.8)	<0.001
Body mass index, mean (SD)	27.5 (3.7)	28.1 (4.1)	28.6 (4.3)	28.9 (4.4)	29.2 (4.4)	<0.001
Diabetes, *n* (%)	62 (14.9)	71 (16.6)	128 (30.4)	152 (30.3)	103 (37.6)	<0.001
*β*-Blockers, %	245 (59.0)	273 (63.9)	366 (87.4)	368 (80.7)	254 (92.7)	<0.001
ACE inhibitors/sartans, %	190 (45.8)	203 (47.5)	330 (78.8)	351 (77.0)	244 (89.1)	<0.001
ACE inhibitors, %	190 (45.8)	203 (47.5)	312 (74.5)	302 (66.2)	205 (74.8)	<0.001
Sartans, %	0 (0.0)	0 (0.0)	22 (5.3)	52 (12.5)	39 (14.2)	<0.001
Calcium antagonists, %	119 (28.7)	142 (33.3)	88 (21.0)	94 (20.6)	76 (27.7)	0.01
Diuretics, %	71 (17.1)	86 (21.1)	134 (32.1)	163 (35.8)	113 (41.2)	<0.001
Other, %	2 (0.5)	9 (2.1)	10 (2.4)	31 (6.8)	8 (2.9)	<0.001
Number of blood pressure lowering drugs, mean (SD)	1.51 (0.92)	1.67 (1.00)	2.22 (0.89)	2.23 (1.04)	2.54 (0.83)	<0.001

CABG, coronary artery bypass grafting; PCI, percutaneous coronary intervention.

**Table 2:** Time trends in blood pressure and proportions of patients with blood pressure at recommended goal (crude values).

	Survey I *n* = 415	Survey II *n* = 427	Survey III *n* = 421	Survey IV *n* = 456	Survey V *n* = 274	*p* for trend
Systolic blood pressure, mean (SD)	137.9 (21.4)	139.5 (21.6)	136.1 (20.3)	134.8 (22.0)	134.2 (18.6)	<0.001
Diastolic blood pressure, mean (SD)	83.4 (11.0)	84.8 (12.0)	85.2 (11.0)	80.9 (12.5)	81.1 (10.4)	<0.001
Mean arterial pressure, mean (SD)	101.5 (12.6)	103.1 (13.9)	102.2 (12.8)	98.9 (14.3)	98.8 (12.1)	<0.001
Pulse pressure, mean (SD)	54.5 (18.1)	54.6 (16.0)	50.9 (15.8)	54.0 (16.4)	53.1 (13.8)	0.17
Blood pressure at recommended goal,^*∗*^*n* (%)	204 (49.2)	190 (44.5)	188 (44.7)	233 (51.1)	161 (58.8)	0.007
Blood pressure <140/90 mmHg, *n* (%)	223 (53.7)	212 (49.7)	225 (53.4)	262 (57.5)	167 (60.9)	0.01

^*∗*^BP goal of <140/90 mmHg (<130/80 mmHg in diabetics) in 1997–1998, 1999–2000, 2006–2007, and 2011–2013; <140/90 mmHg (<140/85 mmHg in diabetics) in 2016–2017.

**Table 3:** Time trends in least squares means of blood pressure according to sex, age, and education. Multivariable models include age, sex, education, employment, index event, and body mass index.

	Survey I	Survey II	Survey III	Survey IV	Survey V	*p* for trend	*p* for interaction
Systolic blood pressure							
Men, mean (SD)	138.2 (20.2)	138.5 (22.9)	134.0 (19.7)	134.2 (19.6)	132.1 (20.5)	<0.001	0.12
Women, mean (SD)	140.2 (22.2)	144.1 (22.2)	139.9 (22.0)	134.0 (22.2)	134.9 (22.7)	<0.001	

Diastolic blood pressure, mean (SD)							
Men, mean (SD)	83.9 (11.7)	84.3 (11.4)	85.4 (11.4)	81.8 (11.4)	81.8 (11.9)	<0.001	0.10
Women, mean (SD)	81.6 (11.9)	85.9 (11.9)	84.3 (11.8)	79.0 (11.9)	80.7 (12.1)	<0.05	

Systolic blood pressure							
Age ≤60 years, mean (SD)	135.4 (19.7)	134.3 (19.3)	129.1 (19.4)	130.7 (19.4)	134.5 (19.7)	0.06	0.13
Age >60 years, mean (SD)	142.3 (19.8)	145.4 (19.5)	141.4 (19.6)	136.7 (19.5)	134.2 (19.8)	<0.001	

Diastolic blood pressure, mean (SD)							
Age ≤60 years, mean (SD)	84.8 (12.0)	84.9 (11.8)	85.3 (11.8)	82.2 (11.8)	84.5 (12.0)	0.18	0.35
Age >60 years, mean (SD)	81.8 (11.4)	84.9 (11.4)	84.9 (11.3)	79.8 (11.3)	80.1 (11.9)	<0.001	

Systolic blood pressure							
Vocational or primary education, mean (SD)	140.7 (22.3)	141.4 (21.8)	138.6 (21.6)	136.0 (21.8)	134.0 (22.4)	<0.01	0.87
At least secondary education, mean (SD)	137.3 (19.4)	138.5 (19.2)	132.8 (19.0)	132.1 (19.1)	133.1 (19.9)	<0.001	

Diastolic blood pressure, mean (SD)							
Vocational or primary education, mean (SD)	83.5 (10.6)	84.5 (11.2)	86.5 (11.6)	81.7 (12.2)	82.0 (16.1)	0.11	0.40
At least secondary education, mean (SD)	82.9 (11.1)	85.1 (11.0)	83.7 (10.8)	80.3 (10.9)	81.0 (11.4)	<0.001	

**Table 4:** Odds ratios of blood pressure at goal by surveys. Multivariable models include age, sex, education, employment, index event, and body mass index.

Survey	Odds ratio (95% confidence intervals)	
	Blood pressure at recommended goal^*∗*^	Blood pressure <140/90 mmHg
Survey I	1.0	1.0
Survey II	0.89 (0.67–1.18)	0.91 (0.69–1.20)
Survey III	0.90 (0.67–1.27)	1.06 (0.79–1.42)
Survey IV	1.43 (1.06–1.93)	1.43 (1.06–1.92)
Survey V	1.89 (1.29–2.74)	1.63 (1.12–2.38)
*p* for trend	<0.001	<0.001

^*∗*^BP goal of <140/90 mmHg (<130/80 mmHg in diabetics) in 1997–1998, 1999–2000, 2006–2007, and 2011–2013; <140/90 mmHg (<140/85 mmHg in diabetics) in 2016–2017.

## Data Availability

Data are available upon request.
